# The frequency of *NPM1 *mutations in childhood acute myeloid leukemia

**DOI:** 10.1186/1756-8722-3-41

**Published:** 2010-10-27

**Authors:** Maria Braoudaki, Chrissa Papathanassiou, Katerina Katsibardi, Natalia Tourkadoni, Kalliopi Karamolegou, Fotini Tzortzatou-Stathopoulou

**Affiliations:** 1 University Research Institute for the Study and Treatment of Childhood Genetic and Malignant Diseases, University of Athens, "Aghia Sophia" Children's Hospital, Athens, Greece; 2 Hematology/Oncology Unit, First Department of Pediatrics, University of Athens, "Aghia Sophia" Children's Hospital, Athens, Greece

## Abstract

**Background:**

Mutations in the nucleophosmin *(NPM1) *gene have been solely associated with childhood acute myeloid leukemia (AML). We evaluated the frequency of *NPM1 *mutations in childhood AML, their relation to clinical and cytogenetic features and the presence of common *FLT3 *and *RAS *mutations.

**Results:**

*NPM1 *mutations were found in 8% of cases. They involved the typical type 'A' mutation and one novel mutation characterized by two individual base pair substitutions, which resulted in 2 amino acid changes (W290) and (S293) in the NPM protein. *FLT3*/ITD mutations were observed in 12% of the cases and in one *NPM1-*mutated case bearing also t(8;21) (q22;q22). No common *RAS *mutations were identified.

**Conclusions:**

A relatively consistent *NPM1 *mutation rate was observed, but with variations in types of mutations. The role of different types of *NPM1 *mutations, either individually or in the presence of other common gene mutations may be essential for childhood AML prognosis.

## Background

Acute myeloid leukemia (AML) is a genetically and phenotypically heterogenous disease that accounts for 15-20% of childhood leukemia [1]. Several genetic mutations, gene rearrangements and chromosomal translocations are involved in the pathogenesis of leukemia. Chromosomal abnormalities like the t(15;17) or the inv(16) have been associated with a particular morphology and clinical behavior [2]. However, in patients with no detectable chromosomal abnormalities, the genetic background remains unknown [3,4]. Conversely, previous work has indicated the involvement of various gene mutations with prognostic relevance in AML, including activating mutations of genes encoding transcription factors (*AML1, CEBPα*), tyrosine kinases (*FLT3*, *KIT*) or their downstream effectors (*NRAS*) and nucleophosmin (*NPM1*) mutations [3,5].

Nucleophosmin is a multifunctional nucleocytoplasmic protein involved in several cellular activities, such as ribosomal biosynthesis, maintenance of genome stability and molecular chaperone functions [6,7]. Abnormal expression of NPM may lead to the oncogenesis of some types of leukemia as *NPM1 *gene is a partner in several tumor associated chromosomal translocations [5]. A number of studies have described the presence of common mutations within the final exon (exon-12) of the *NPM1 *gene in patients with AML [1,5,7-11]. These mutations cause the cytoplasmic localization of NPM and abrogate its function [12].

*NPM1 *gene mutations have been described in both adult and pediatric patients with variable prevalence and proven to have prognostic significance. *NPM1 *is mutated in a large proportion (30-50%) of adult AML cases with a normal karyotype [8,13]. This subset of AML patients that exhibit a normal karyotype account for approximately 50% of cases and thus far have a markedly variable outcome. The *NPM1 *mutations in AML cases with a normal karyotype have been significantly associated with high frequency of internal tandem duplications of FMS-like tyrosine kinase-3 (*FLT3/I*TD) [1], which are considered to confer a less favorable prognosis.

The current study was undertaken to evaluate the prevalence of *NPM1 *mutations in childhood AML in association with cytogenetic analysis, molecular screening of common gene mutations and patients' clinical characteristics, in order to address its prognostic relevance.

## Methods

### Patient Samples

A total of 28 pediatric patients were diagnosed with AML within a 10-year period. The patient population comprised primarily of Greek children (24/28), whilst the rest of the cohort included Albanian (3/28) and Romanian (1/28) patients. All patients received chemotherapy according to BFM AML protocol (BFM87; n = 14 and BFM04; n = 11) for 12 months. Patient samples were obtained from bone marrow aspirates at diagnosis. Sufficient amount of DNA for analysis of *NPM1 *mutations was available in 25/28 (89.3%) patients at diagnosis. Of those, 18/25 were diagnosed with *de novo *AML and 7/25 with secondary AML following myelodysplastic syndrome (MDS). The patients' median age was 7 years (range 1-14 years) and among them, 12/28 (48%) patients were male. The diagnosis was based on the French-American-British (FAB) classification scheme and immunophenotype. The study population included 1 patient with M0, 4 patients with M1, 4 patients with M2, 3 patients with M4, 5 patients with M5 (4M5a and 1M5b) and 1 patient with M6 FAB subtype. This study was approved by the Medical School of the University of Athens in Greece.

### Cytogenetic analysis

Cytogenetic investigations were performed by karyotyping G-banding analysis in all patients. Additionally, interphase fluorescence in situ hybridization (iFISH) was used to monitor chromosomal aberrations.

### Molecular analyses of *NPM1*, *FLT3 *and *RAS *mutations

Genomic DNA was extracted from bone marrow samples according to the standard phenol-chloroform protocol. The exon 12 of the *NPM1 *gene was amplified using polymerase chain reaction (PCR). The primers and the procedure were adapted from Döhner *et al*. [14]. Mutational analyses of the *FLT3/*AL (activation loop) at positions D835/I836, *FLT3*/ITD and *RAS *genes *(NRAS, HRAS and KRAS) *were performed as previously described [15].

### DNA sequencing

Direct sequencing of both strands of each PCR product was carried out on an ABI PRISM 3100-Avant Genetic Analyser (Applied Biosystems, Foster City, CA), according to the manufacturer's instructions. All samples were sequenced, including those that did not provide preliminary evidence for *FLT3 *mutations based on electrophoresis.

### Statistical analyses

The prevalence of *NPM1 *mutations in AML was too low to permit statistical analysis for correlation with survival. Actuarial estimates of the event-free-survival (EFS) and overall survival (OS) at 5-years were calculated for 20/25 patients (5/25 newly diagnosed) using the Kaplan-Meier method. Event-free-survival is defined as the time from randomisation to treatment failure (relapse, second malignancy or remission failure) or death. Overall survival denotes the percentage of patients survived for a certain period of time since diagnosis or treatment completion. Statistical significance between *NPM1-*wild type and *NPM1*-mutated groups with clinical and cytogenetic characteristics was determined by Fischer's exact test.

## Results

### Patients Characteristics

The laboratory and clinical characteristics between the *NPM1*-mutated group and the *NPM1-*wild type group of patients were compared. The *NPM1 *mutations were present in patients with AML M1 and M2 FAB subtypes. There was no significant difference in the prevalence of *NPM1 *mutations between sexes. In addition, the mutations were not particularly associated with higher white blood cell count (WBC) or increased blast percentage. However, there was a significant difference with regard to age. Τhe median age in *NPM1*-mutated group was 10.5 years and in *NPM1*-unmutated group was 6.5 years (*p *= < 0.001). The study of possible ethnic differences related to the disease was not feasible, due to limited number of patients.

### Cytogenetic analysis

In this study, chromosomal aberrations were observed in 12/25 (48%) cases. In 4/12 (33.3%) patients t(8;21) (q22;q22) was detected, which was principally associated with the AML M2 FAB subtype (75%). This chromosomal abnormality occurred predominantly in children older than 3 years of age (18.2%) and in 16% of the whole AML population. *MLL *gene rearrangements with chromosome 11q23 abnormality were detected in 3/12 (25%) cases; one AML M4 and one M5 newly diagnosed patient with t(9;11)(p22;q23) and one M4 with t(6;11)(q27;q23). The *MLL *gene rearrangements were more common in children younger than 3 years of age (2/3, 66.7%). No *NPM1 *mutations were found in cases with positive *MLL *gene rearrangements.

### Molecular analysis of gene mutations

*NPM1 *gene mutations were detected in 2/25 (8%) patients with AML (2/18 patients were *de novo *AML; one M1 AML and one M2 AML newly diagnosed). One of the *NPM1 *mutations involved multiple base pair substitutions rather than the common 4 base pair insertions. More specifically, the patient acquired a T→G mutation at codon 290, which resulted in a substitution of tryptophan 290 for glycine (W290) and a T→C mutation at codon 293, which resulted in a substitution of serine 293 for proline (S293). This patient also carried a t(8;21) (q22;q22) chromosomal abnormality. The other case involved a type 'A' mutation; a 4-base pair insertion at position nucleotide 960 (Table 1). In our study, there was no significant difference in the frequency of the *NPM1 *mutations in the AML cases with a normal karyotype (7.7%) compared to cases with abnormal karyotype (8.3%). Of note, a normal karyotype was detected in 13/25 (52%) of the AML cases.

**Table 1:** Patients' molecular and clinical characteristics.

**Patient No**.	Nucleotide sequences	Sex	Age (years)	FAB Type	Karyotype	*MLL *rearrangement	*FLT3 *mutation	WBC	Blast Count in BM (%)	Survival
Wild type	gat ctc tgg cag tgg agg aag tct ctt taa gaa aat ag									
1	gat ctc tg**t ctg**gca gtg gag gaa gtc tct tta aga aaa tag	M	8 years	M1	46, xy	N	None	23900	85%	Complete Remission
2	gat ctc tgg cag **g**gg agg aag **c**ct ctt taa gaa aat ag	F	13 years	M2	46, xx t(8;21) (q22;q22)	N	*FLT3*/ITD	7680	60%	Complete Remission

### Analysis of *NPM1 *mutations compared to *FLT3 *and *RAS *mutations

All cases were analyzed for *FLT3/ITD *and *FLT3/*AL mutations, whereas only the two *NPM1 *mutant cases were screened for *NRAS, KRAS *and *HRAS *mutations. No common *RAS *mutations among the *NPM1*-mutated cases were observed. Overall, *FLT3*/ITD mutations were found in 3/25 (12%) of AML patients (2/3 newly diagnosed). Of these, 1 patient also had an *NPM1 *mutation. No *FLT3/*AL mutations were detected.

### EFS & OS

**Figure 1:**
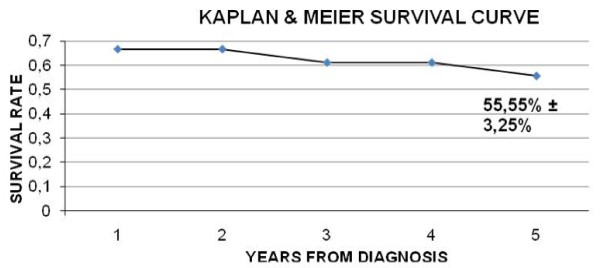
**Kaplan and Meier Survival curve**.

The EFS and OS at 5-years were estimated at 55.55% (SE ± 3.25%) (Figure 1) and 61.70% (SE ± 4.1%), respectively. Comparison between the *NPM1*-mutated group and the *NPM1*-wild type group was not feasible, since the *NPM1 *mutated group was composed of only 2 cases, one of which was newly diagnosed.

## Discussion

The current study attempted to assess the incidence and prognostic relevance of *NPM1 *mutations in childhood AML. *NPM1 *mutations were found in patients with *de novo *AML M1 and M2 subtypes. No mutations were observed in patients with AML M5 FAB subtypes, which comprised the larger group in this study. Previous studies in childhood AML also suggested absence of *NPM1 *mutations in M5 cases [2,16]. In concurrence with other reports [1,4,7], there was no significant association between *NPM1 *mutations and sex, high WBC or increased blast percentage in the bone marrow at diagnosis.

*NPM1 *mutations were found in patients above 3 years of age. This is in agreement with previous studies that have also demonstrated a trend towards higher probability of *NPM1 *mutations for older AML pediatric patients [1,4,17]. Rau and Brown [17] proposed the possibility of a relative myeloid progenitor cell resistance to *NPM1 *mutations in younger pediatric patients.

In our study, t(8;21)(q22;q22) was observed in 16% of the total AML cases and in 33.3% of the cases bearing a chromosomal aberration. *NPM1 *mutations were observed in one M2 AML case bearing a t(8;21)(q22;q22). Previous studies suggested that in AML, especially in the M2 subtype, translocation t(8;21)(q22;q22) is one of the most frequent chromosomal abnormalities and can be found in 5-12% of AML cases [18].

Frequently, translocations involving chromosome 11q23 can be found in 15-20% of pediatric AML cases and are, in general, associated with a poor outcome [19]. In line with other work [1], our study demonstrated that translocations involving *MLL *gene rearrangements with chromosome 11q23 abnormality occurred in 12% of patients and was more common in children younger than 3 years of age (66.6%).

Progression of MDS to AML may represent a similar, though, more complicated model for leukemic transformation [20]. In the current study, no *NPM1 *mutations were detected in cases with secondary AML following MDS, which is in line with previous studies associating absence or low rates of *NPM1 *mutations in patients with MDS [10,21].

Mutations of the *NPM1 *gene were present in 8% of AML cases in this study. This is in agreement with previous reports on childhood AML [1,4,17]. More than 40 different types of *NPM1 *mutations have been detected, with types A, B and D being the most common [7]. In our study, sequencing analysis confirmed the presence of a type 'A' mutation in one *NPM1*-mutated case. The majority of *NPM1 *mutations encode mutant proteins that have a novel nuclear export signal (NES) motif inserted at the C-terminus and are thought to play a significant role in the abnormal cytoplasmic localization of the NPM protein. The other mutation obtained in the present study, involved 2 individual base pair substitutions which resulted in 2 amino acid changes (W290) and (S293) in the NPM protein. To our knowledge, this is a novel mutation, even though disruption of the nucleolar localization signal (NLS) at C-terminus due to mutations in the tryptophan residue 290 has been previously described [17]. More specifically, the tryptophan residue at position 290 is considered essential to the nucleolar localization of the NPM protein [2], however, the overall impact of the presence of both amino acid changes that were detected in our study, remains undefined.

*FLT3 *gene mutations were identified in 12% of the total AML cases. This is in line with other studies, in which 11.5% of the cases carried an ITD mutation in the *FLT3 *gene [4]. *FLT3*/ITD mutation was observed in one *NPM1-*mutated case bearing t(8;21) (q22;q22). It is not feasible to predict the prognostic value of both mutations in the presence of this translocation, since the time this patient has been monitored is rather short. Rau and Brown [17] and Boonthimat *et al*. [22] suggested a principal prevalence of *FLT3*/ITD mutations in *NPM1*-mutated cases, due to a possible pathogenic link between these two gene mutations.

No correlation was found between *RAS *mutations and the frequency of *NPM1 *mutations. This was similarly observed by Boonthimat *et al*. [22] who suggested that *NPM1 *and *RAS *do not cooperate in the pathogenic model of AML. Of note, *NRAS *mutations are normally found in AML cases with inv(16), which are essentially mutually exclusive of *NPM1 *mutations [23].

To conclude, it seems that *NPM1 *mutations are consistently present in approximately 10% of childhood AML cases [17]. However the observation of a high variety of *NPM1 *mutations merits further studies, in order to determine their individual contribution to the pathogenesis of childhood AML and their comprehensible relation to prognosis.

## Competing interests

The authors declare that they have no competing interests.

## Authors' contributions

MB organized the research plan, analyzed data, performed experiments and drafted the paper. CP and KK, carried out part of the experiments. TN and KK provided samples and clinical data and F.T-S coordinated the study, participated in its design and contributed to writing. All authors read and approved the final manuscript.
